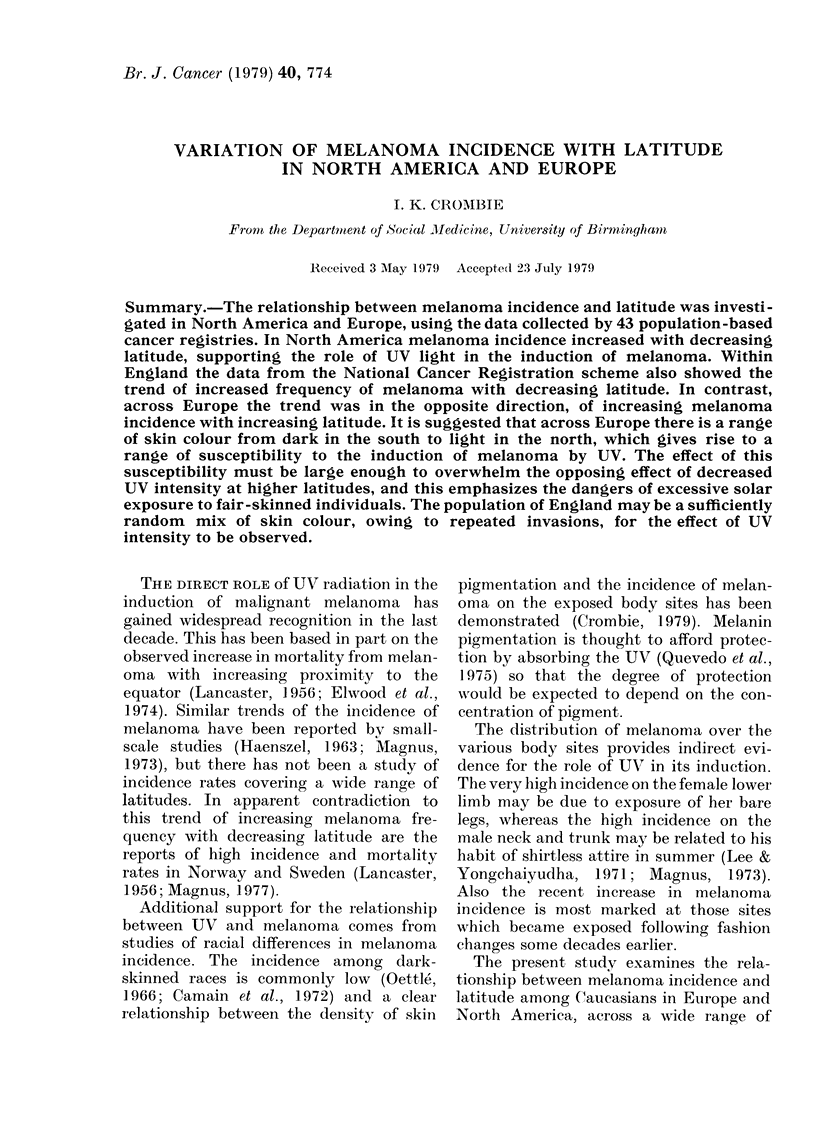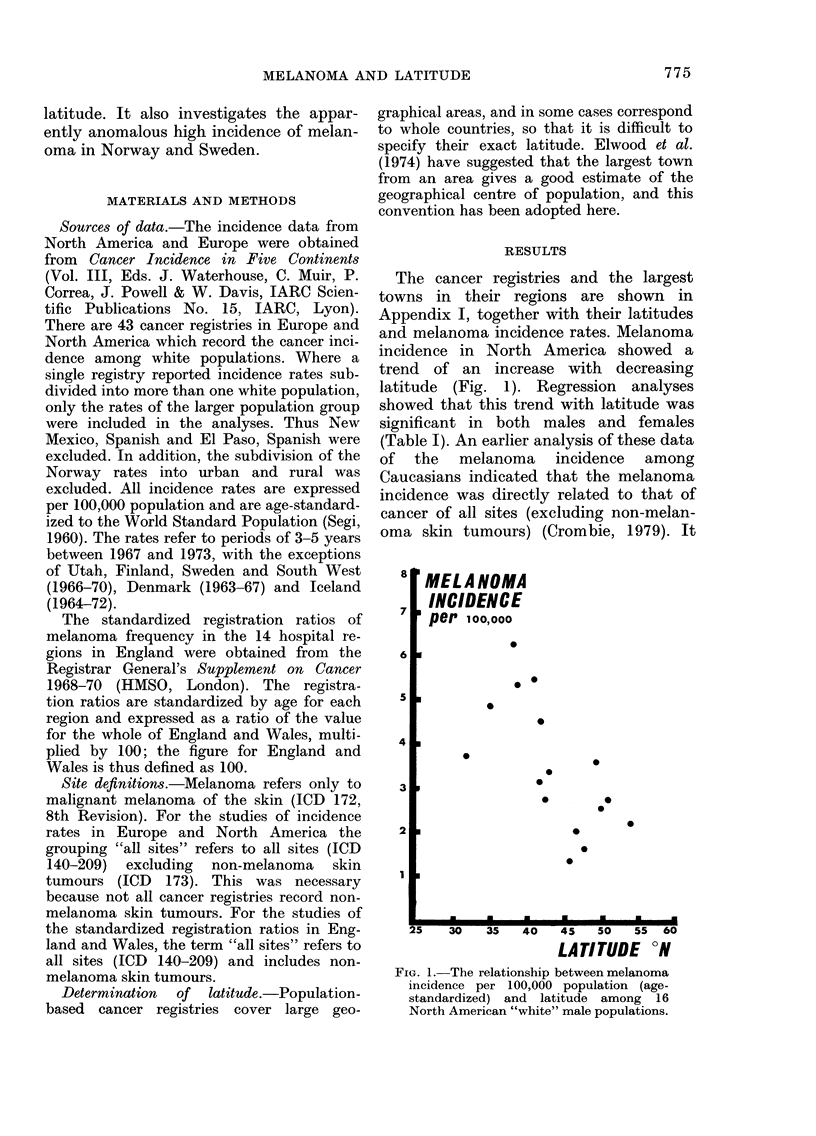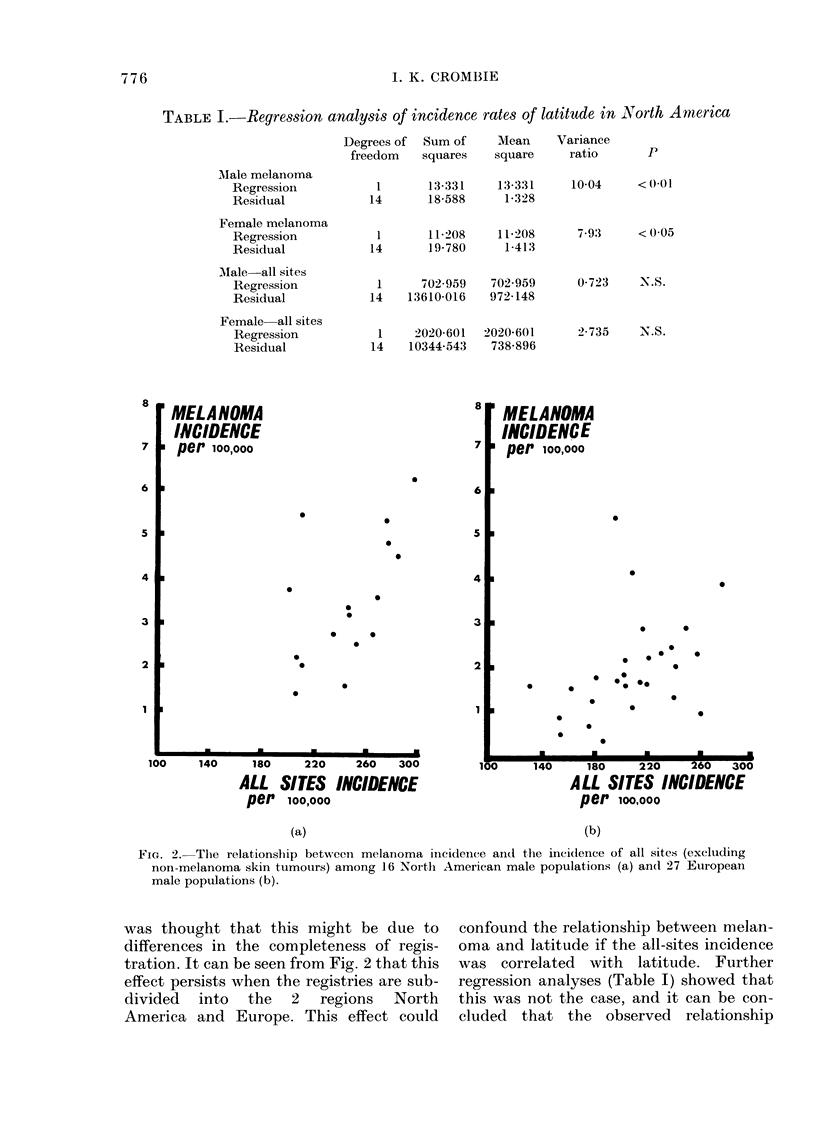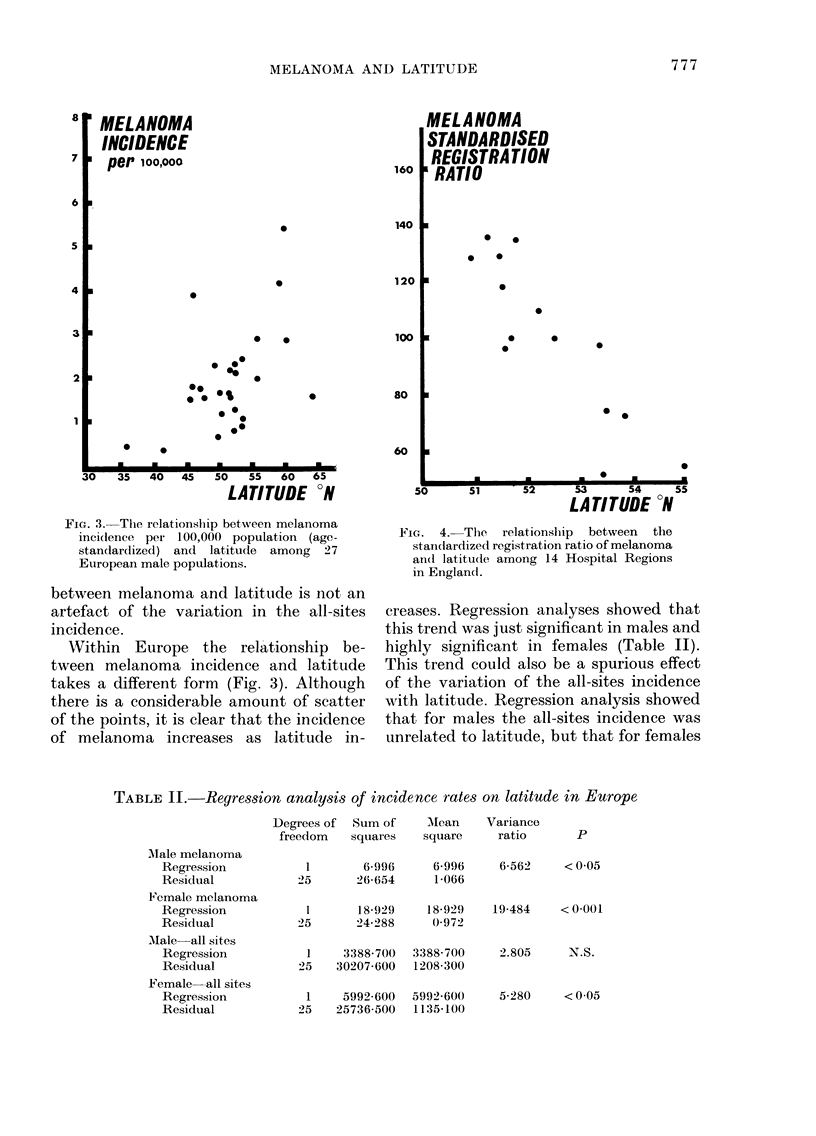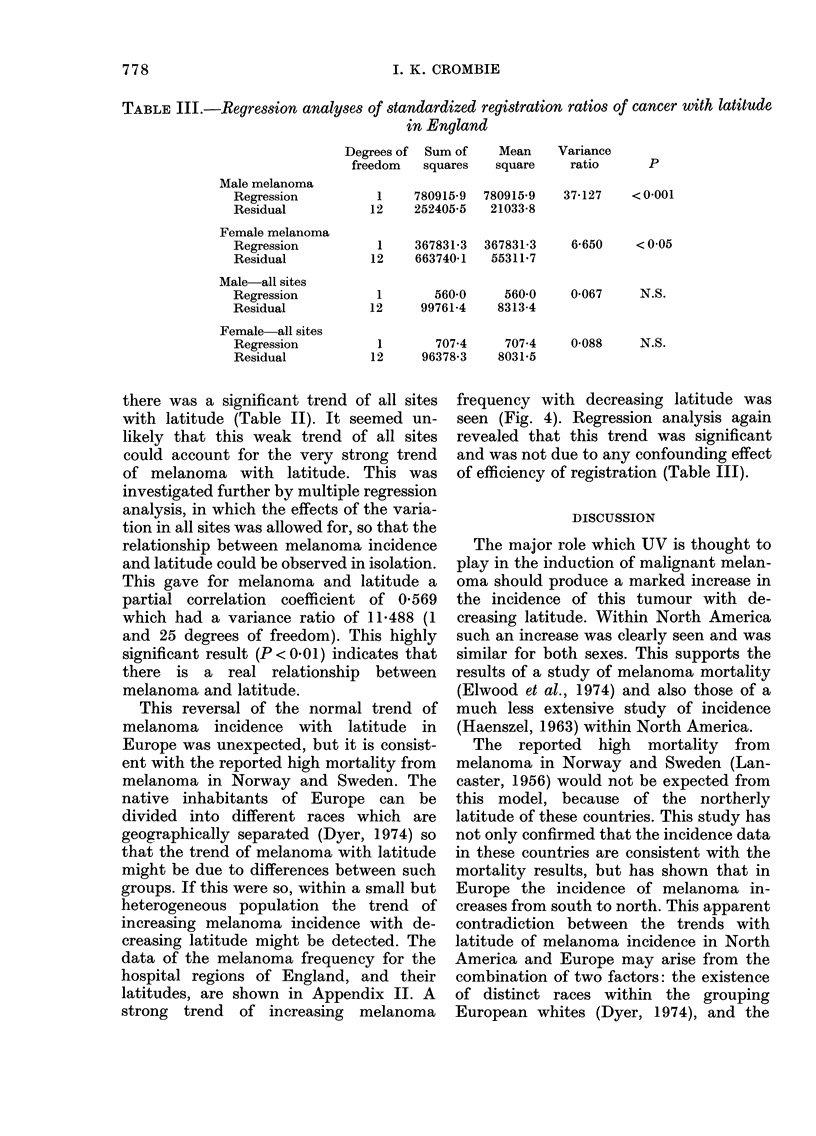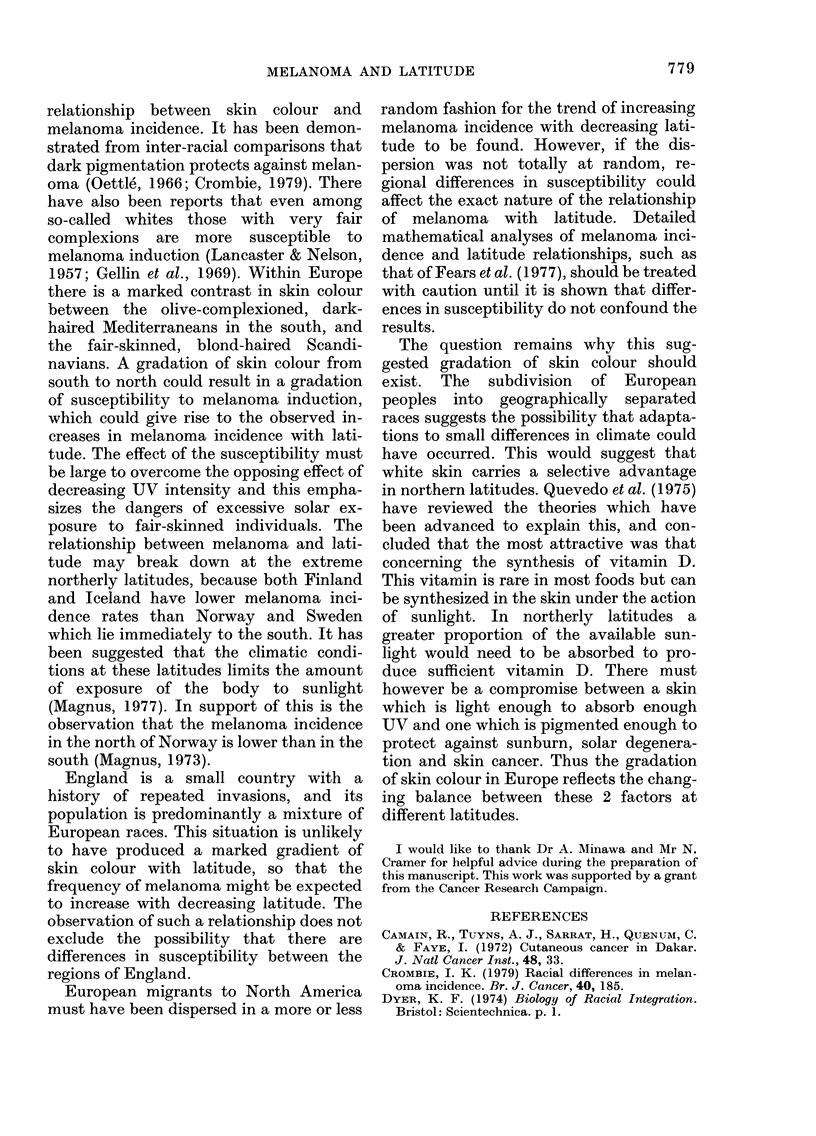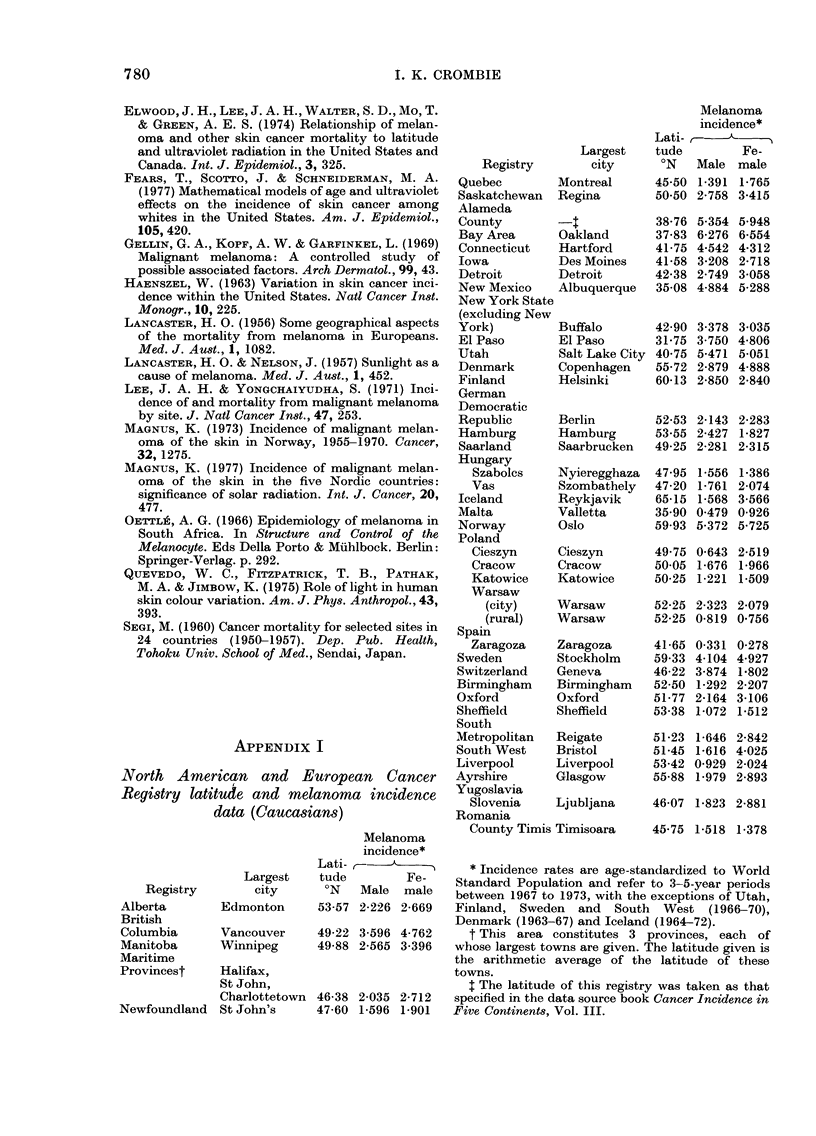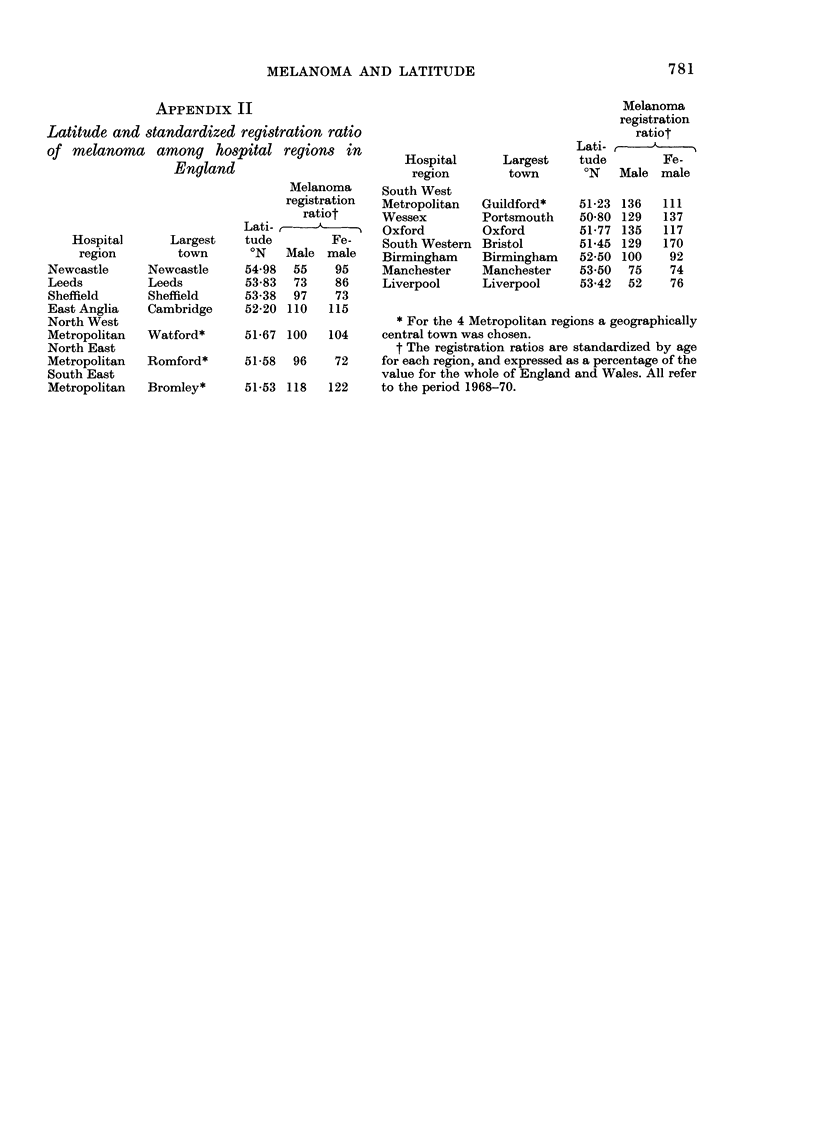# Variation of melanoma incidence with latitude in North America and Europe.

**DOI:** 10.1038/bjc.1979.260

**Published:** 1979-11

**Authors:** I. K. Crombie

## Abstract

The relationship between melanoma incidence and latitude was investigated in North American and Europe, using the data collected by 43 population-based cancer registries. In North America melanoma incidence increased with decreasing latitude, supporting the role of UV light in the induction of melanoma. Within England the data from the National Cancer Registration scheme also showed that trend of decreased frequency of melanoma with decreasing latitude. In contrast, across Europe the trend was in the opposite direction, of increasing melanoma incidence with increasing latitude. It is suggested that across Europe there is a range of skin colour from dark in the south to light in the north, which gives rise to a range of susceptibility to the induction of melanoma by UV. The effect of this susceptibility must be large enough to overwhelm the opposing effect of decreased UV intensity at higher latitudes, and this emphasizes the dangers of excessive solar exposure to fair-skinned individuals. The populations of England may be a sufficiently random mix of skin colour, owing to repeated invasions, for the effect of UV intensity to be observed.


*Br. J. Cancer* (1979) **40**, 774

### VARIATION OF MELANOMA INCIDENCE WITH LATITUDE IN NORTH AMERICA AND EUROPE

I. K. CROMBIE

*From the Department of Social Medicine, University of Birmingham*

Received 3 May 1979 Accepted 23 July 1979

**Summary.—The relationship between melanoma incidence and latitude was investigated in North America and Europe, using the data collected by 43 population-based cancer registries. In North America melanoma incidence increased with decreasing latitude, supporting the role of UV light in the induction of melanoma. Within England the data from the National Cancer Registration scheme also showed the trend of increased frequency of melanoma with decreasing latitude. In contrast, across Europe the trend was in the opposite direction, of increasing melanoma incidence with increasing latitude. It is suggested that across Europe there is a range of skin colour from dark in the south to light in the north, which gives rise to a range of susceptibility to the induction of melanoma by UV. The effect of this susceptibility must be large enough to overwhelm the opposing effect of decreased UV intensity at higher latitudes, and this emphasizes the dangers of excessive solar exposure to fair-skinned individuals. The population of England may be a sufficiently random mix of skin colour, owing to repeated invasions, for the effect of UV intensity to be observed.**

THE DIRECT ROLE of UV radiation in the induction of malignant melanoma has gained widespread recognition in the last decade. This has been based in part on the observed increase in mortality from melanoma with increasing proximity to the equator (Lancaster, 1956; Elwood *et al.*, 1974). Similar trends of the incidence of melanoma have been reported by small-scale studies (Haenszel, 1963; Magnus, 1973), but there has not been a study of incidence rates covering a wide range of latitudes. In apparent contradiction to this trend of increasing melanoma frequency with decreasing latitude are the reports of high incidence and mortality rates in Norway and Sweden (Lancaster, 1956; Magnus, 1977).

Additional support for the relationship between UV and melanoma comes from studies of racial differences in melanoma incidence. The incidence among dark-skinned races is commonly low (Oettlé, 1966; Camain *et al.*, 1972) and a clear relationship between the density of skin pigmentation and the incidence of melanoma on the exposed body sites has been demonstrated (Crombie, 1979). Melanin pigmentation is thought to afford protection by absorbing the UV (Quevedo *et al.*, 1975) so that the degree of protection would be expected to depend on the concentration of pigment.

The distribution of melanoma over the various body sites provides indirect evidence for the role of UV in its induction. The very high incidence on the female lower limb may be due to exposure of her bare legs, whereas the high incidence on the male neck and trunk may be related to his habit of shirtless attire in summer (Lee & Yongchaiyudha, 1971; Magnus, 1973). Also the recent increase in melanoma incidence is most marked at those sites which became exposed following fashion changes some decades earlier.

The present study examines the relationship between melanoma incidence and latitude among Caucasians in Europe and North America, across a wide range of latitude. It also investigates the apparently anomalous high incidence of melanoma in Norway and Sweden.

MATERIALS AND METHODS

*Sources of data.*—The incidence data from North America and Europe were obtained from *Cancer Incidence in Five Continents* (Vol. III, Eds. J. Waterhouse, C. Muir, P. Correa, J. Powell & W. Davis, IARC Scientific Publications No. 15, IARC, Lyon). There are 43 cancer registries in Europe and North America which record the cancer incidence among white populations. Where a single registry reported incidence rates subdivided into more than one white population, only the rates of the larger population group were included in the analyses. Thus New Mexico, Spanish and El Paso, Spanish were excluded. In addition, the subdivision of the Norway rates into urban and rural was excluded. All incidence rates are expressed per 100,000 population and are age-standardized to the World Standard Population (Segi, 1960). The rates refer to periods of 3–5 years between 1967 and 1973, with the exceptions of Utah, Finland, Sweden and South West (1966–70), Denmark (1963–67) and Iceland (1964–72).

The standardized registration ratios of melanoma frequency in the 14 hospital regions in England were obtained from the Registrar General's *Supplement on Cancer* 1968–70 (HMSO, London). The registration ratios are standardized by age for each region and expressed as a ratio of the value for the whole of England and Wales, multiplied by 100; the figure for England and Wales is thus defined as 100.

*Site definitions.*—Melanoma refers only to malignant melanoma of the skin (ICD 172, 8th Revision). For the studies of incidence rates in Europe and North America the grouping "all sites" refers to all sites (ICD 140–209) excluding non-melanoma skin tumours (ICD 173). This was necessary because not all cancer registries record non-melanoma skin tumours. For the studies of the standardized registration ratios in England and Wales, the term "all sites" refers to all sites (ICD 140–209) and includes non-melanoma skin tumours.

*Determination of latitude.*—Population-based cancer registries cover large geographical areas, and in some cases correspond to whole countries, so that it is difficult to specify their exact latitude. Elwood *et al.* (1974) have suggested that the largest town from an area gives a good estimate of the geographical centre of population, and this convention has been adopted here.

RESULTS

The cancer registries and the largest towns in their regions are shown in Appendix I, together with their latitudes and melanoma incidence rates. Melanoma incidence in North America showed a trend of an increase with decreasing latitude (Fig. 1). Regression analyses showed that this trend with latitude was significant in both males and females (Table I). An earlier analysis of these data of the melanoma incidence among Caucasians indicated that the melanoma incidence was directly related to that of cancer of all sites (excluding non-melanoma skin tumours) (Crombie, 1979). It

FIG. 1.—The relationship between melanoma incidence per 100,000 population (age-standardized) and latitude among 16 North American "white" male populations.

TABLE I.—*Regression analysis of incidence rates of latitude in North America*

| | Degrees of freedom | Sum of squares | Mean square | Variance ratio | P |
|---|---|---|---|---|---|
| Male melanoma | | | | | |
| Regression | 1 | 13·331 | 13·331 | 10·04 | < 0·01 |
| Residual | 14 | 18·588 | 1·328 | | |
| Female melanoma | | | | | |
| Regression | 1 | 11·208 | 11·208 | 7·93 | < 0·05 |
| Residual | 14 | 19·780 | 1·413 | | |
| Male—all sites | | | | | |
| Regression | 1 | 702·959 | 702·959 | 0·723 | N.S. |
| Residual | 14 | 13610·016 | 972·148 | | |
| Female—all sites | | | | | |
| Regression | 1 | 2020·601 | 2020·601 | 2·735 | N.S. |
| Residual | 14 | 10344·543 | 738·896 | | |

FIG. 2.—The relationship between melanoma incidence and the incidence of all sites (excluding non-melanoma skin tumours) among 16 North American male populations (a) and 27 European male populations (b).

was thought that this might be due to differences in the completeness of registration. It can be seen from Fig. 2 that this effect persists when the registries are subdivided into the 2 regions North America and Europe. This effect could confound the relationship between melanoma and latitude if the all-sites incidence was correlated with latitude. Further regression analyses (Table I) showed that this was not the case, and it can be concluded that the observed relationship

FIG. 3.—The relationship between melanoma incidence per 100,000 population (age-standardized) and latitude among 27 European male populations.

FIG. 4.—The relationship between the standardized registration ratio of melanoma and latitude among 14 Hospital Regions in England.

between melanoma and latitude is not an artefact of the variation in the all-sites incidence.

Within Europe the relationship between melanoma incidence and latitude takes a different form (Fig. 3). Although there is a considerable amount of scatter of the points, it is clear that the incidence of melanoma increases as latitude increases. Regression analyses showed that this trend was just significant in males and highly significant in females (Table II). This trend could also be a spurious effect of the variation of the all-sites incidence with latitude. Regression analysis showed that for males the all-sites incidence was unrelated to latitude, but that for females

TABLE II.—*Regression analysis of incidence rates on latitude in Europe*

| | Degrees of freedom | Sum of squares | Mean square | Variance ratio | $P$ |
|---|---|---|---|---|---|
| Male melanoma | | | | | |
| Regression | 1 | 6·996 | 6·996 | 6·562 | $<0·05$ |
| Residual | 25 | 26·654 | 1·066 | | |
| Female melanoma | | | | | |
| Regression | 1 | 18·929 | 18·929 | 19·484 | $<0·001$ |
| Residual | 25 | 24·288 | 0·972 | | |
| Male—all sites | | | | | |
| Regression | 1 | 3388·700 | 3388·700 | 2.805 | N.S. |
| Residual | 25 | 30207·600 | 1208·300 | | |
| Female—all sites | | | | | |
| Regression | 1 | 5992·600 | 5992·600 | 5·280 | $<0·05$ |
| Residual | 25 | 25736·500 | 1135·100 | | |

TABLE III.—*Regression analyses of standardized registration ratios of cancer with latitude in England*

| | Degrees of freedom | Sum of squares | Mean square | Variance ratio | $P$ |
|---|---|---|---|---|---|
| Male melanoma | | | | | |
| Regression | 1 | 780915·9 | 780915·9 | 37·127 | $<0·001$ |
| Residual | 12 | 252405·5 | 21033·8 | | |
| Female melanoma | | | | | |
| Regression | 1 | 367831·3 | 367831·3 | 6·650 | $<0·05$ |
| Residual | 12 | 663740·1 | 55311·7 | | |
| Male—all sites | | | | | |
| Regression | 1 | 560·0 | 560·0 | 0·067 | N.S. |
| Residual | 12 | 99761·4 | 8313·4 | | |
| Female—all sites | | | | | |
| Regression | 1 | 707·4 | 707·4 | 0·088 | N.S. |
| Residual | 12 | 96378·3 | 8031·5 | | |

there was a significant trend of all sites with latitude (Table II). It seemed unlikely that this weak trend of all sites could account for the very strong trend of melanoma with latitude. This was investigated further by multiple regression analysis, in which the effects of the variation in all sites was allowed for, so that the relationship between melanoma incidence and latitude could be observed in isolation. This gave for melanoma and latitude a partial correlation coefficient of 0·569 which had a variance ratio of 11·488 (1 and 25 degrees of freedom). This highly significant result ($P < 0·01$) indicates that there is a real relationship between melanoma and latitude.

This reversal of the normal trend of melanoma incidence with latitude in Europe was unexpected, but it is consistent with the reported high mortality from melanoma in Norway and Sweden. The native inhabitants of Europe can be divided into different races which are geographically separated (Dyer, 1974) so that the trend of melanoma with latitude might be due to differences between such groups. If this were so, within a small but heterogeneous population the trend of increasing melanoma incidence with decreasing latitude might be detected. The data of the melanoma frequency for the hospital regions of England, and their latitudes, are shown in Appendix II. A strong trend of increasing melanoma frequency with decreasing latitude was seen (Fig. 4). Regression analysis again revealed that this trend was significant and was not due to any confounding effect of efficiency of registration (Table III).

### DISCUSSION

The major role which UV is thought to play in the induction of malignant melanoma should produce a marked increase in the incidence of this tumour with decreasing latitude. Within North America such an increase was clearly seen and was similar for both sexes. This supports the results of a study of melanoma mortality (Elwood *et al.*, 1974) and also those of a much less extensive study of incidence (Haenszel, 1963) within North America.

The reported high mortality from melanoma in Norway and Sweden (Lancaster, 1956) would not be expected from this model, because of the northerly latitude of these countries. This study has not only confirmed that the incidence data in these countries are consistent with the mortality results, but has shown that in Europe the incidence of melanoma increases from south to north. This apparent contradiction between the trends with latitude of melanoma incidence in North America and Europe may arise from the combination of two factors: the existence of distinct races within the grouping European whites (Dyer, 1974), and the relationship between skin colour and melanoma incidence. It has been demonstrated from inter-racial comparisons that dark pigmentation protects against melanoma (Oettlé, 1966; Crombie, 1979). There have also been reports that even among so-called whites those with very fair complexions are more susceptible to melanoma induction (Lancaster & Nelson, 1957; Gellin *et al.*, 1969). Within Europe there is a marked contrast in skin colour between the olive-complexioned, dark-haired Mediterraneans in the south, and the fair-skinned, blond-haired Scandinavians. A gradation of skin colour from south to north could result in a gradation of susceptibility to melanoma induction, which could give rise to the observed increases in melanoma incidence with latitude. The effect of the susceptibility must be large to overcome the opposing effect of decreasing UV intensity and this emphasizes the dangers of excessive solar exposure to fair-skinned individuals. The relationship between melanoma and latitude may break down at the extreme northerly latitudes, because both Finland and Iceland have lower melanoma incidence rates than Norway and Sweden which lie immediately to the south. It has been suggested that the climatic conditions at these latitudes limits the amount of exposure of the body to sunlight (Magnus, 1977). In support of this is the observation that the melanoma incidence in the north of Norway is lower than in the south (Magnus, 1973).

England is a small country with a history of repeated invasions, and its population is predominantly a mixture of European races. This situation is unlikely to have produced a marked gradient of skin colour with latitude, so that the frequency of melanoma might be expected to increase with decreasing latitude. The observation of such a relationship does not exclude the possibility that there are differences in susceptibility between the regions of England.

European migrants to North America must have been dispersed in a more or less random fashion for the trend of increasing melanoma incidence with decreasing latitude to be found. However, if the dispersion was not totally at random, regional differences in susceptibility could affect the exact nature of the relationship of melanoma with latitude. Detailed mathematical analyses of melanoma incidence and latitude relationships, such as that of Fears *et al.* (1977), should be treated with caution until it is shown that differences in susceptibility do not confound the results.

The question remains why this suggested gradation of skin colour should exist. The subdivision of European peoples into geographically separated races suggests the possibility that adaptations to small differences in climate could have occurred. This would suggest that white skin carries a selective advantage in northern latitudes. Quevedo *et al.* (1975) have reviewed the theories which have been advanced to explain this, and concluded that the most attractive was that concerning the synthesis of vitamin D. This vitamin is rare in most foods but can be synthesized in the skin under the action of sunlight. In northerly latitudes a greater proportion of the available sunlight would need to be absorbed to produce sufficient vitamin D. There must however be a compromise between a skin which is light enough to absorb enough UV and one which is pigmented enough to protect against sunburn, solar degeneration and skin cancer. Thus the gradation of skin colour in Europe reflects the changing balance between these 2 factors at different latitudes.

I would like to thank Dr A. Minawa and Mr N. Cramer for helpful advice during the preparation of this manuscript. This work was supported by a grant from the Cancer Research Campaign.

### REFERENCES

CAMAIN, R., TUYNS, A. J., SARRAT, H., QUENUM, C. & FAYE, I. (1972) Cutaneous cancer in Dakar. *J. Natl Cancer Inst.*, **48**, 33.

CROMBIE, I. K. (1979) Racial differences in melanoma incidence. *Br. J. Cancer*, **40**, 185.

DYER, K. F. (1974) *Biology of Racial Integration*. Bristol: Scientechnica. p. 1.

Elwood, J. H., Lee, J. A. H., Walter, S. D., Mo, T. & Green, A. E. S. (1974) Relationship of melanoma and other skin cancer mortality to latitude and ultraviolet radiation in the United States and Canada. *Int. J. Epidemiol.*, **3**, 325.

Fears, T., Scotto, J. & Schneiderman, M. A. (1977) Mathematical models of age and ultraviolet effects on the incidence of skin cancer among whites in the United States. *Am. J. Epidemiol.*, **105**, 420.

Gellin, G. A., Kopf, A. W. & Garfinkel, L. (1969) Malignant melanoma: A controlled study of possible associated factors. *Arch Dermatol.*, **99**, 43.

Haenszel, W. (1963) Variation in skin cancer incidence within the United States. *Natl Cancer Inst. Monogr.*, **10**, 225.

Lancaster, H. O. (1956) Some geographical aspects of the mortality from melanoma in Europeans. *Med. J. Aust.*, **1**, 1082.

Lancaster, H. O. & Nelson, J. (1957) Sunlight as a cause of melanoma. *Med. J. Aust.*, **1**, 452.

Lee, J. A. H. & Yongchaiyudha, S. (1971) Incidence of and mortality from malignant melanoma by site. *J. Natl Cancer Inst.*, **47**, 253.

Magnus, K. (1973) Incidence of malignant melanoma of the skin in Norway, 1955–1970. *Cancer*, **32**, 1275.

Magnus, K. (1977) Incidence of malignant melanoma of the skin in the five Nordic countries: significance of solar radiation. *Int. J. Cancer*, **20**, 477.

Oettlé, A. G. (1966) Epidemiology of melanoma in South Africa. In *Structure and Control of the Melanocyte*. Eds Della Porto & Mühlbock. Berlin: Springer-Verlag. p. 292.

Quevedo, W. C., Fitzpatrick, T. B., Pathak, M. A. & Jimbow, K. (1975) Role of light in human skin colour variation. *Am. J. Phys. Anthropol.*, **43**, 393.

Segi, M. (1960) Cancer mortality for selected sites in 24 countries (1950–1957). *Dep. Pub. Health, Tohoku Univ. School of Med.*, Sendai, Japan.

### Appendix I

*North American and European Cancer Registry latitude and melanoma incidence data (Caucasians)*

| Registry | Largest city | Latitude °N | Melanoma incidence* Male | Female |
|---|---|---|---|---|
| Alberta | Edmonton | 53·57 | 2·226 | 2·669 |
| British Columbia | Vancouver | 49·22 | 3·596 | 4·762 |
| Manitoba | Winnipeg | 49·88 | 2·565 | 3·396 |
| Maritime Provinces† | Halifax, St John, Charlottetown | 46·38 | 2·035 | 2·712 |
| Newfoundland | St John's | 47·60 | 1·596 | 1·901 |
| Quebec | Montreal | 45·50 | 1·391 | 1·765 |
| Saskatchewan | Regina | 50·50 | 2·758 | 3·415 |
| Alameda County | —‡ | 38·76 | 5·354 | 5·948 |
| Bay Area | Oakland | 37·83 | 6·276 | 6·554 |
| Connecticut | Hartford | 41·75 | 4·542 | 4·312 |
| Iowa | Des Moines | 41·58 | 3·208 | 2·718 |
| Detroit | Detroit | 42·38 | 2·749 | 3·058 |
| New Mexico | Albuquerque | 35·08 | 4·884 | 5·288 |
| New York State (excluding New York) | Buffalo | 42·90 | 3·378 | 3·035 |
| El Paso | El Paso | 31·75 | 3·750 | 4·806 |
| Utah | Salt Lake City | 40·75 | 5·471 | 5·051 |
| Denmark | Copenhagen | 55·72 | 2·879 | 4·888 |
| Finland | Helsinki | 60·13 | 2·850 | 2·840 |
| German Democratic Republic | Berlin | 52·53 | 2·143 | 2·283 |
| Hamburg | Hamburg | 53·55 | 2·427 | 1·827 |
| Saarland | Saarbrucken | 49·25 | 2·281 | 2·315 |
| Hungary | | | | |
| Szabolcs | Nyiereggháza | 47·95 | 1·556 | 1·386 |
| Vas | Szombathely | 47·20 | 1·761 | 2·074 |
| Iceland | Reykjavik | 65·15 | 1·568 | 3·566 |
| Malta | Valletta | 35·90 | 0·479 | 0·926 |
| Norway | Oslo | 59·93 | 5·372 | 5·725 |
| Poland | | | | |
| Cieszyn | Cieszyn | 49·75 | 0·643 | 2·519 |
| Cracow | Cracow | 50·05 | 1·676 | 1·966 |
| Katowice | Katowice | 50·25 | 1·221 | 1·509 |
| Warsaw | | | | |
| (city) | Warsaw | 52·25 | 2·323 | 2·079 |
| (rural) | Warsaw | 52·25 | 0·819 | 0·756 |
| Spain | | | | |
| Zaragoza | Zaragoza | 41·65 | 0·331 | 0·278 |
| Sweden | Stockholm | 59·33 | 4·104 | 4·927 |
| Switzerland | Geneva | 46·22 | 3·874 | 1·802 |
| Birmingham | Birmingham | 52·50 | 1·292 | 2·207 |
| Oxford | Oxford | 51·77 | 2·164 | 3·106 |
| Sheffield | Sheffield | 53·38 | 1·072 | 1·512 |
| South Metropolitan | Reigate | 51·23 | 1·646 | 2·842 |
| South West | Bristol | 51·45 | 1·616 | 4·025 |
| Liverpool | Liverpool | 53·42 | 0·929 | 2·024 |
| Ayrshire | Glasgow | 55·88 | 1·979 | 2·893 |
| Yugoslavia | | | | |
| Slovenia | Ljubljana | 46·07 | 1·823 | 2·881 |
| Romania | | | | |
| County Timis | Timisoara | 45·75 | 1·518 | 1·378 |

* Incidence rates are age-standardized to World Standard Population and refer to 3–5-year periods between 1967 to 1973, with the exceptions of Utah, Finland, Sweden and South West (1966–70), Denmark (1963–67) and Iceland (1964–72).

† This area constitutes 3 provinces, each of whose largest towns are given. The latitude given is the arithmetic average of the latitude of these towns.

‡ The latitude of this registry was taken as that specified in the data source book *Cancer Incidence in Five Continents*, Vol. III.

### APPENDIX II

*Latitude and standardized registration ratio of melanoma among hospital regions in England*

| Hospital region | Largest town | Latitude °N | Melanoma registration ratio† Male | Female |
|---|---|---|---|---|
| Newcastle | Newcastle | 54·98 | 55 | 95 |
| Leeds | Leeds | 53·83 | 73 | 86 |
| Sheffield | Sheffield | 53·38 | 97 | 73 |
| East Anglia | Cambridge | 52·20 | 110 | 115 |
| North West Metropolitan | Watford* | 51·67 | 100 | 104 |
| North East Metropolitan | Romford* | 51·58 | 96 | 72 |
| South East Metropolitan | Bromley* | 51·53 | 118 | 122 |
| South West Metropolitan | Guildford* | 51·23 | 136 | 111 |
| Wessex | Portsmouth | 50·80 | 129 | 137 |
| Oxford | Oxford | 51·77 | 135 | 117 |
| South Western | Bristol | 51·45 | 129 | 170 |
| Birmingham | Birmingham | 52·50 | 100 | 92 |
| Manchester | Manchester | 53·50 | 75 | 74 |
| Liverpool | Liverpool | 53·42 | 52 | 76 |

* For the 4 Metropolitan regions a geographically central town was chosen.

† The registration ratios are standardized by age for each region, and expressed as a percentage of the value for the whole of England and Wales. All refer to the period 1968–70.